# Feasibility of a Virtual Reality System in Speech Therapy: From Assessment to Tele-Rehabilitation in Children with Cerebral Palsy

**DOI:** 10.3390/children11111327

**Published:** 2024-10-30

**Authors:** Gloria Mangani, Veronica Barzacchi, Clara Bombonato, Jessica Barsotti, Elena Beani, Valentina Menici, Carolina Ragoni, Giuseppina Sgandurra, Benedetta Del Lucchese

**Affiliations:** 1 Developmental Neurology and Neurorehabilitation Unit, IRCCS Stella Maris Foundation, 56126 Pisa, Italy; gloria.mangani@fsm.unipi.it (G.M.); veronica.barzacchi@fsm.unipi.it (V.B.); clara.bombonato@fsm.unipi.it (C.B.); jessica.barsotti@fsm.unipi.it (J.B.); elena.beani@fsm.unipi.it (E.B.); valentina.menici@fsm.unipi.it (V.M.); carolina.ragoni@fsm.unipi.it (C.R.); benedetta.dellucchese@fsm.unipi.it (B.D.L.); 2 Tuscan Ph.D. Programme of Neuroscience, University of Florence, 50121 Florence, Italy; 3 Department of Clinical and Experimental Medicine, University of Pisa, 56126 Pisa, Italy; 4 Ph.D. Programme in Clinical and Translational Sciences, University of Pisa, 56126 Pisa, Italy

**Keywords:** neurodevelopmental disorders, language abilities, cerebral palsy, technology, learning abilities, language rehabilitation, learning rehabilitation

## Abstract

Background/Objectives: New advances in technologies are opening the possibility to support functional evaluation and rehabilitation in the field of speech therapy. Among available systems, a virtual reality rehabilitation system (VRRS, Khymeia) is a multi-domain ecosystem. Despite it being used in a limited number of studies, its use in speech-therapy has shown potential for promoting linguistic and literacy skills. Methods: This pilot study aims to assess the feasibility of single-session speech assessment with the VRRS in twenty-eight children with cerebral palsy (CP) by means of ad hoc questionnaires. Moreover, we evaluated the feasibility and the effects of an intensive tele-rehabilitation treatment with the VRRS in a subgroup of three children with unilateral CP. Results: Feasibility was generally good when using the VRRS for assessments. Both clinicians and children found it to have good usability, although acceptability scores were higher for children than clinicians. For tele-rehabilitation, overall improvements were observed in both linguistic and learning (reading and writing) skills. Conclusions: This study paves the way for VRRS use in speech-therapy tele-rehabilitation for children with CP and language and learning difficulties.

## 1. Introduction

Cerebral palsy (CP) is one of the most common causes of developmental disability. It is defined as a group of permanent movement and posture disorders that are attributed to non-progressive disturbances that occur during fetal or infant brain development, causing activity limitations [1]. Beyond neuromotor deficits as core symptoms of CP, the complexity and variability of clinical expression of this disorder is now well recognized [2], with associated disorders including cognitive, sensory-perceptual, language, and communication impairments [3]. Specifically, communication difficulties have been reported in the literature, with a variable prevalence from 40% [4] to 61% [5]. These impairments can appear in different ways, including atypical development of gestures and facial expressions, as well as receptive and expressive language difficulties, with multiple levels of disrupted speech, involving the development of sounds (articulation), the rules of sound combinations (phonology), phonatory support, and the precise execution of speech movements (dysarthria) and their planning/programming (apraxia of speech), that can reduce reduced speech intelligibility [6]. Mei et al., 2015 [5] examined the language profile of children with CP aged 5 to 6 years, identifying language difficulties in 61% of cases. Among these, 44% exhibited impairments in both expressive and receptive components, while isolated receptive (7%) and expressive (5%) impairments occurred relatively infrequently. Deficits were described across language subdomains (i.e., semantics, syntax, morphology), rather than in single domains. In terms of expressive language subdomains, all verbal participants showed semantic deficits, with 87% showing difficulties in language structure, particularly in syntax and morphology. However, the majority demonstrated appropriate abilities in expressive vocabulary (96%) and spatial concepts (83%). In contrast, when examining receptive language skills, among the 27 verbal participants with language impairment, Mei et al., 2015 [5] observed that comprehension of syntactic structures was the most frequently affected area (93%), while 48% of the sample showed affected morphological structures. Receptive semantic errors also commonly involved comprehension of quantitative concepts (96%) and vocabulary (70%). Additionally, 24% of the sample consisted of non-verbal children. Considering language impairments as potential risk factors for difficulties in education, studies investigating the prevalence of specific learning disorders (SLDs) in children with CP estimated that up to 50% of children with CP may be affected by one or more SLDs in areas not necessarily linked to language (e.g., mathematics) [7]. The widespread impact of such comorbidities that affect educational outcomes, social support, peer relationships, and overall participation in daily life [8] could be reduced by early and accurate functional assessments combined with timely, targeted interventions [9]. Research evidence confirms the positive effect of speech and language therapy on children with CP [10], implementing speech abilities (such as improving intelligibility and speech motor control) [6,11] and overall communication skills, such as expressive or receptive language together with dialogical and interaction abilities to maximize children’s ability to communicate [10]. Speech and language treatment can be administered in a variety of settings, such as children’s homes, hospitals, and schools, employing both direct (i.e., directed to the child) and indirect (i.e., directed to familiar communication partners, such as family members, teachers, and teaching assistants; the objective is to change the way these partners communicate with the child in order to help the child develop communication skills) intervention modalities and individual and group settings [10]. In recent years, the application of new technologies in healthcare, especially after the COVID-19 pandemic, has opened up new possibilities in the assessment and tele-rehabilitation of language functions (e.g., receptive and expressive morphosyntactic and lexical abilities, phonological accuracy and awareness, as well as sentence and period construction) [12,13] and learning skills (reading and writing abilities) [14,15,16,17] in the pediatric population. This innovative approach can in fact be both cost- and time-effective [18] while offering a playful and motivating rehabilitation setting with tailored activities [19], monitored by therapists both online and offline. During online sessions, clinicians can control the technology remotely and interact with the patient in real time, while offline sessions are scheduled by clinicians beforehand. In offline sessions, the patient does the assigned tasks on their own, and the clinician can use the session report generated to monitor the patient’s progress [20]. These new technologies in fact often output quantitative reports, which can serve as additional information to be integrated with traditional assessments and provide a more complete and detailed overview of patient progress. However, there are only a few studies that explore the feasibility and effectiveness of the use of new technologies in the field of speech therapy for children with CP [13]. One such new technology is virtual reality (VR). As a computer-based artificial environment simulating real-life scenarios, it can promote enhanced learning by utilizing sensory feedback (i.e., auditory, visual, and tactile) and user-friendly, enriched environments [21]. VR interventions have been increasingly employed in the rehabilitation of different neurological and neuropsychological dysfunctions [22], mainly in adulthood [23,24,25], though some promising results have been observed in children [26].

In this context, the virtual reality rehabilitation system (VRRS) created by Khymeia (Padua, Italy) stands out as a promising tool for rehabilitation. This comprehensive and integrated system supports various rehabilitation domains, including neurological, orthopedic, cognitive, speech, cardiorespiratory, and postural therapy. While primarily validated in the adult population, there are potential applications in the treatment of neurodevelopmental disorders.

A narrative review by Macchitella et al. (2023) [22] outlines a limited number of studies on the application of the VRRS in speech therapy [13,27,28,29], with just two studies focusing on the pediatric population [13,27]. Cappadona et al. (2023) [13] demonstrated that VRRS can be a valuable tool for implementing effective speech rehabilitation in children with developmental language disorders, while Maresca et al. (2022) [27] reported a significant improvements in both word-reading test scores and homophonic writing in children with dyslexia after treatment with VRRS. In this framework, this study aims to assess the feasibility of the VRRS in speech therapy assessment in children with CP and to explore preliminary findings about tele-rehabilitation protocols in a smaller sample, describing some qualitative results concerning intervention efficacy.

This work was conducted as part of a study aimed to implement a national tele-rehabilitation network across Italy to share a large amount of data on issues and barriers to the use of technological devices in the pediatric population, allowing for investigating their applicability in traditional clinical practice to ensure continuity of care through standardized protocols.

## 2. Materials and Methods

### 2.1. Participants

In total, twenty-eight children (18 males and 10 females, mean age 8.42 ± 2.36 years) were recruited among children and adolescents with CP, referring to IRCCS Fondazione Stella Maris (Pisa) for multidimensional evaluation. Three of the twenty-eight children (all males; mean age 7.74 ± 0.65 years) also completed a tele-rehabilitation intervention. Participants were required to meet the following inclusion criteria: (a) CP diagnosis, (b) no severe comorbid medical conditions, (c) a cognitive functioning that allows for a full understanding of game instructions and adequate collaboration, (d) level of manual abilities according to the Manual Ability Classification System (MACS) [30] < 5, (e) level of communication abilities according to Communication Function Classification System (CFCS) [31] ≤ 3.

Within the recruited sample, 90% of children were diagnosed with spastic CP, while 10% were diagnosed with dyskinetic CP. A total of 75% of the children were CFCS level I, 11% were CFCS level II, and 14% were CFCS level III.

Table 1 shows a detailed descriptive analysis of the sample, including demographic and clinical variables, such as age, sex, diagnosis, cognitive, motor, and communication functions. In particular, children were classified according to CFCS, MACS, and Gross Motor Function Classification System (GMFCS) [32]. Intellectual functioning was also rated using standardized assessments (WISC IV [33], WPPSI III or IV [34], Leiter-3 [35]).

### 2.2. Ethical Approval

This study was approved by The Pediatric Ethics Section of Tuscany Regional Ethics Committee (Italy) with opinion registration number 221/2020.

This study was also registered in the Clinical Trial Protocol Registry and Results System (ClinicalTrials.gov, accessed on code: NCT06290297).

Parents and children signed a written consent form (for children, in a child-friendly format).

### 2.3. Study Procedure

All participants completed a single hour-long VRRS one-shot assessment consisting of a set of selected speech, language, and learning activities, as part of the multidimensional functional assessment (a detailed description of the device is provided below). Two different protocols were created and delivered according to children’s learning abilities: a preschooler protocol—where reading and writing skills were not required—and a school-age protocol. Ad hoc feasibility questionnaires were completed by the main stakeholders (participants and the clinicians who administered the tasks) at the end of the session. After the one-shot assessment, three of the twenty-eight recruited children started an intensive speech-therapy tele-rehabilitation intervention with VRRS. The intervention was planned by the clinician based on language and learning goals highlighted from the multidimensional functional evaluation and according to the feasibility assessment performed.

Criteria for starting the training included both children’s and their family’s compliance with tele-rehabilitation, the availability of a suitable setting, and a good Internet connection. Activities within the device library were selected and personalized by the clinician according to the children’s needs and goals. The activities were made increasingly more complex as the children’s performance improved. 

The 3 months of training were paired with a motivational framework through a PowerPoint presentation, tailored to each child’s interests, in order to achieve greater motivation and compliance as well as visualizing tele-rehabilitation progression (e.g., number of sessions completed and missed). For this study, among the available VRRS options, the VRRS Home Kit Tablet was used for speech-therapy assessment and tele-rehabilitation. 

#### 2.3.1. Intervention

After a familiarization phase, with clinicians explaining how the system works to children and caregivers while in in-patient care, the VRRS Home Kit was sent to the participants’ homes along with a printed instruction manual. Children were asked to complete the allocated 30 to 45 min long sessions 3 times a week for 3 months. All sessions were tailored to each participant’s rehabilitation goals and needs, choosing specific language exercises activities and personalizing different options. To improve metaphonological abilities, as well as reading, writing, and lexical language skills, particular activities were delivered during the intervention. In the initial phase of the tele-rehabilitation pathway, clinicians provided online supervision in two sessions per week. During these sessions, clinicians engaged with patients in real time through a dedicated system and were able to remotely control the tablet. As participants gained more confidence, the number of online sessions gradually decreased in favor of more independent, offline sessions, where children completed pre-scheduled training sessions on their own. Clinicians closely tracked treatment progress through a specialized web platform and changed the exercises of the session every two/three weeks, following the child’s improvement. 

Pre-post assessments were administered the week before (T0) and after (T1) the tele-rehabilitation training.

#### 2.3.2. VRRS Home Kit Tablet

**Figure 1:**
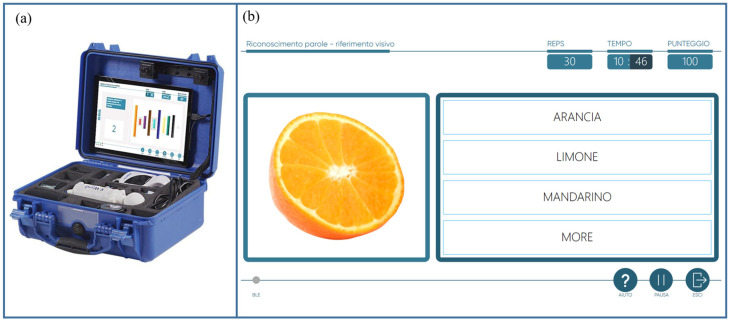
(**a**) VRRS Home Kit Tablet: A case provided to patients and their families, equipped with a tablet and a set of integrated motion sensors, allowing for home tele-rehabilitation pathways. (**b**) Example of language domain’ exercise available within the wide library of the medical device.

The VRRS is developed by the Italian company Khymeia and offers a variety of non-immersive virtual reality devices. It integrates multi-domain technology designed for rehabilitation in both clinical and home-based settings. Utilizing biofeedback and augmented feedback, the system is designed to be user-friendly and engaging [36,37]. Initially developed for adult patients, VRRS has recently been adapted for children and adolescents, with recent studies showing promising results [38,39,40,41]. Among the VRRS devices, the Home Kit Tablet (Figure 1) allows for cognitive, language, and motor rehabilitation activities to be delivered at home through tele-rehabilitation pathways. This system includes a tablet with an extensive library of exercises, alongside different USB-connected peripherals such as balance boards and sensors, designed for motor rehabilitation. Additionally, touch-screen activities enable neuropsychological and speech-therapy sessions.

The VRRS speech-therapy domain exercises are designed to assess and enhance language and learning skills. Specifically, for language and speech, the VRRS includes activities targeting both receptive and expressive abilities within the morpho-syntactic and semantic–lexical domains. In terms of learning skills, the system offers exercises focused on reading, writing, and phonological awareness. All exercises can be customized: clinicians are able to change difficulty levels and configure other exercise-specific settings (e.g., duration, speed).

### 2.4. Outcome Measures

All patients underwent a usability and acceptability questionnaire. Participants also completed an exhaustive speech and language assessment performed by expert clinicians before (T0) and after (T1) the tele-rehabilitation period. Standardized tests are detailed below.

#### 2.4.1. Feasibility Measures

The feasibility was examined through ad hoc purpose-designed questionnaires, according to standard items identified in the literature regarding the Technology Assessment Model (TAM) [42] and conforming to the definition of usability [43,44,45] and acceptability [46]. The questionnaires were completed by the main stakeholders after the assessment (clinicians and children) and after training (clinicians, children and caregivers). In the children’s questionnaires, questions were structured to be easy to understand for even the youngest participant).

Two questionnaires were designed: an assessment questionnaire and a training questionnaire.

Assessment questionnaires were filled out by both the clinician who administered the evaluation and the child. Only children whose cognitive functioning allowed them to fully understand the questions were required to answer.

Both the clinicians’ and children’s questionnaires included an introductory section providing information about the child, the device, and the assessment completed.

The questionnaire contained 16 questions, which were ranked on a 5-point Likert scale (1 most negative, 5 most positive) and were split into usability (e.g., from clinicians’ assessment questionnaire: “Did you manage to achieve the goals you had set at the beginning?”) and acceptability (e.g., from children’ assessment questionnaire: “Overall, how easy was it to play with the system?”) domains; each section could range from a minimum of 8 (strongly negative) to a maximum of 40 (strongly positive). Additionally, the children’s questionnaire included a section with questions about the motivation of use (e.g., “Would you like using the system at home for therapy?”), increasing the number of questions from 16 to 18. A smiley meter was used with younger children to help them with answering and expressing their emotions; it featured five illustrated faces with different expressions (from saddest to happiest) and children had to select the face that best represented their feelings.

Beyond multiple-choice questions, participants could add further comments about the experience with VRRS.

Sometimes, participants were requested to provide their personal insights on some open-ended items in order to gain a broader understanding of their feedback.

A four-section 5-point Likert scale training questionnaire was also created to assess training feasibility. As for the assessment, a 5-point Likert scale training questionnaire was also created to assess the training’ feasibility, with four different sections. 

Specifically, four different areas were investigated: (i) system adaptability (whether the system is suitable for delivering tele-rehabilitation in children, e.g., from the clinicians’ training questionnaire: “Overall, how satisfied are you with using the system for home-based training with this patient?”); (ii) exercise customization (their preferences regarding the type of exercise, how the participants felt about the exercises, the perceived level of difficulty, e.g., from the parents’ training questionnaire: “did the child succeed in reaching the activities’ goals?); (iii) required effort (whether participants felt that the treatment was exhausting and demanding; e.g., from the children’s training questionnaire: “How tired did you get during the training?”); (iv) system appropriateness (whether the system is adequate in terms of tablet and software, e.g., from the children’ training questionnaire: “How much did you enjoy using the system as it is?”).

After the training, clinicians, children (when their cognitive abilities allowed for full comprehension of the questions), and parents completed the questionnaires. The children’s and parents’ questionnaires each consisted of 24 items, while the clinicians’ version contained 20 items.

#### 2.4.2. Standardized Language and Academic Skills Assessment

Receptive and expressive vocabulary were assessed through a standardized TNL-Test Neuropsicologico Lessicale [Neuropsychological Lexical Test] [47].

Specifically, receptive vocabulary was assessed by a figured multiple-choice test. Expressive vocabulary was investigated though a rapid figured-naming test and a phonologically and semantically aided facilitated-naming task, composing a raw lexical-naming score (expressive vocabulary score). The raw scores were converted into T-scores according to the test manual. For this study, the raw receptive vocabulary and expressive vocabulary scores (raw lexical-naming score) were considered.

CMF [48] is designed to evaluate metaphonological skills in children aged 5 to 11 years. Metaphonological skills are crucial for the development of reading and writing skills, closely related to language competencies. Several subtests were used to assess different aspects and levels of phonological awareness. In particular, the assessment for preschool children was composed of a segmentation test (identifying and articulating the segmental syllable units in the correct order of the words), a synthesis test (blending the syllable spoken by the examiner in the correct sentence to form a word), discrimination word/non-word pairs (identifying different words based on a single acoustic feature, minimal pairs), and recognizing initial syllables and rhymes (identifying the initial or final syllable of a word).

The CMF school-children protocol consisted of the following tasks: segmentation test (identifying and articulating the phonemes in the correct order that to make up various words), synthesis test (blending a series of phonemes spoken by the examiner in the correct sequence to form a word), deleting the initial or final syllable test (pronouncing a word without its initial or final syllable), and an FAS test (a verbal fluency test with phonemic facilitation, where children must produce as many words as possible starting with the same letter or sound and recognition of rhymes by identifying the final syllable of a word).

The total number of correct answers in each subtest determines the subject’s score.

Standardized test batteries (ALCE Battery, Protocollo di valutazione di lettura e scrittura Martini) for Italian reading ability were used to test the presence of reading difficulties. Reading speed and accuracy were evaluated by presenting children with sets of words and non-words and asking them to read each one. Reading speed was measured in syllables per second (*s*/*s*). The error rate was calculated using the total number of words read.

In particular, the ALCE battery (Assessment Lettura e Comprensione in età Evolutiva [Assessment of Reading and Comprehension skills in Developmental Age] [49] is a standardized test battery for the assessment of reading and comprehension skills in primary school children between first to fifth grade. Words and non-words tasks were administered. Raw scores were converted into T-scores according to the test manual.

The Protocollo di valutazione di lettura e scrittura Martini, 1995 [Reading and Writing Assessment Protocol, Martini] has reading tasks consisting of flat-intonation two- and three-syllable words and two- and three-syllable words with consonant clusters. Raw scores were transformed into standardized z-scores according to normative data. This battery was administered to children with greater reading impairments, and in some cases, considering these, it was not possible to administer all the subtests.

Writing abilities were tested as the accuracy in writing tasks in Protocollo di valutazione di lettura e scrittura Martini [Reading and Writing Assessment Protocol] [50] and DDE-2 [51].

Protocollo di valutazione di lettura e scrittura Martini, 1995 includes writing tasks with lists of two-syllable words featuring flat intonation and consonant clusters, as well as three-syllable words with similar characteristics. This battery was administered to children with severe writing difficulties. The DDE-2 assessment involved lists of both real words and non-words that the child had to write as part of a dictation task. Raw scores were then converted into standardized z-scores based on normative data.

### 2.5. Data Collection

Data on training performance, clinical outcomes, and rating of questionnaires were collected on a digital database available at IRCCS Fondazione Stella Maris.

### 2.6. Statistical Analysis

Clinical data were analyzed using the Statistical Package for Social Sciences (SPSS, version 20.0), considering a *p* < 0.05 as statistically significant. Normality of data distribution was verified by Shapiro–Wilk’s test, and thus parametric analyses were conducted. A *t*-test was used to compare feasibility scores between clinicians and children and among acceptability and usability domains in clinicians. Pearson’s correlation was used to investigate the relation between the clinician’s responses to the feasibility questionnaire, both in terms of usability and acceptability, and the age of the recruited children.

To assess the consistency between responses provided by clinicians and children in the questionnaires, an intraclass correlation (ICC) analysis was conducted using a two-way mixed-effects model. The ICC for consistency was calculated to determine the degree of agreement between the two groups. 

## 3. Results

### 3.1. Feasibility Assessment Outcome

In total, twenty-eight questionnaires compiled by clinicians and twenty-two questionnaires answered by the respective children were analyzed. Six of the twenty-eight recruited children could not respond to the questionnaire due to impaired comprehension skills.

**Figure 2:**
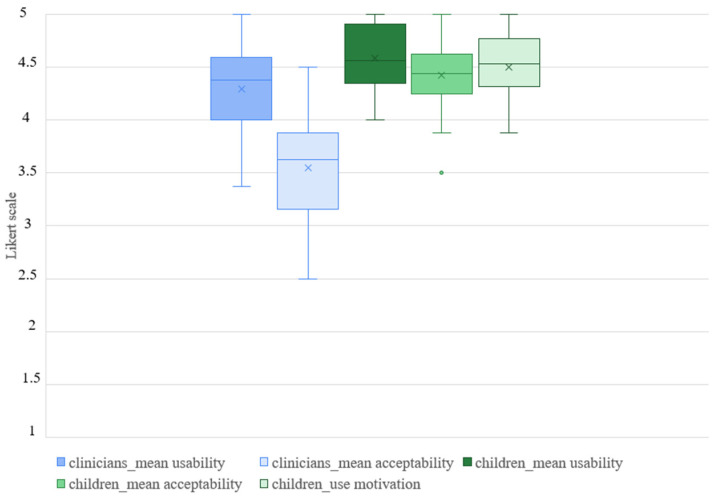
Mean usability and acceptability box-plot of children and clinicians responses to feasibility assessment questionnaires.

Responses to the feasibility assessment questionnaire showed a good level of usability and acceptability from both clinicians (mean usability 4.29 ± SD 0.40; mean acceptability 3.55 ± SD 0.50) and children (mean usability 4.58 ± SD 0.32; mean acceptability 4.42 ± SD 0.33) (see Table 2 and Figure 2).

However, on average, the children’s scores were higher than the clinicians’ scores. This difference is statistically significant in both usability (t = −2.89; *p* < 0.01) and acceptability (t = −6.53; *p* < 0.01) (Table 3).

Statistically significant differences were also observed when comparing clinicians’ usability and acceptability scores (t = 6.11; *p* < 0.01), with higher scores for the usability domain.

A Pearson’s correlation analysis outlined no statistically significant correlation between the age of the children involved in the one-shot assessment with VRRS and clinicians’ usability (r = 0.006) and acceptability (r = −0.106) scores.

A more detailed analysis comparing responses provided by clinicians and children (*n* = 22) revealed a low agreement (ICC = 0.312; CI 95%—0.11–0.642) between the two stakeholders, which was not statistically significant (F = 1.90; *p* > 0.05).

### 3.2. Training Outcome

After the one-shot assessment, three children with unilateral CP took part in an intensive speech-therapy tele-rehabilitation program with VRRS. Two of the children had an average intellectual functioning, while the other had a borderline intellectual functioning. All children were CFCS/GMFCS level I, two were MACS level II, and one was MACS level III.

All participants completed the training with an almost overlapping frequency in three months. They completed an average of 40 sessions (±4) for a mean of 23 (±3.6) h and 37 (±19.3) min.

The tele-rehabilitation intervention had been planned, as children had temporarily stopped their ongoing traditional speech-therapy treatments. At the pre-training assessment, two of the three cases had both receptive and expressive lexical skills in the normal range (T ≥ 50), while one subject had a borderline score in both areas (30 ≤ T ≤ 40).

Significant learning difficulties were observed in all three subjects. Specifically, metaphonological impairments were outlined in T0 and assessed using the school-age protocol for two of the three children and the preschool-age protocol in one, due to the difficulties faced. Significant learning difficulties were observed in all three subjects.

All three subjects showed impaired reading (speed and accuracy) and writing skills, especially one child who could only read and write flat bisyllabic and trisyllabic words.

**Figure 3:**
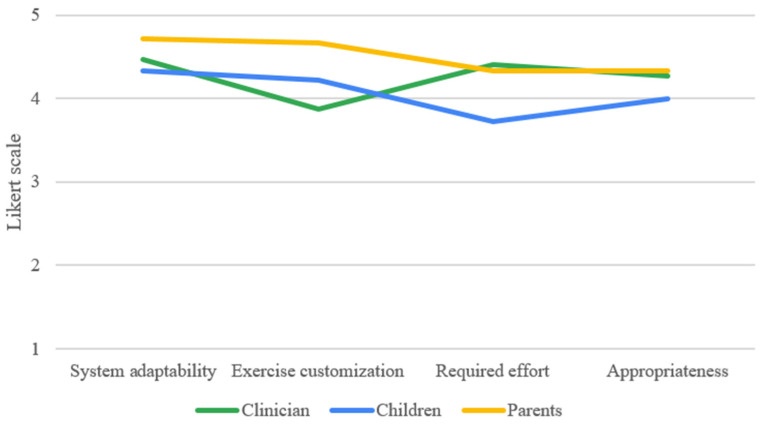
Clinicians, parents, and children responses to feasibility-training questionnaires.

Stakeholders scores (clinicians, parents, and children) post-tele-rehabilitation showed a good level of overall feasibility. As shown in Table 3 and Figure 3, the “system adaptability” section had higher scores in all stakeholders. Clinicians’ responses showed lower scores in the “exercise customization” section (mean 3.87 ± 0.74), as both parents (mean 4.33 ± 0.77) and children (mean 3.72 ± 1.36) showed lower scores in the “required effort” section.

Furthermore, qualitative improvements in language and learning skills were noted in all three subjects at T1 assessment. Due to the limited sample size, it was not possible to conduct pre- and post-treatment statistical analyses.

**Figure 4:**
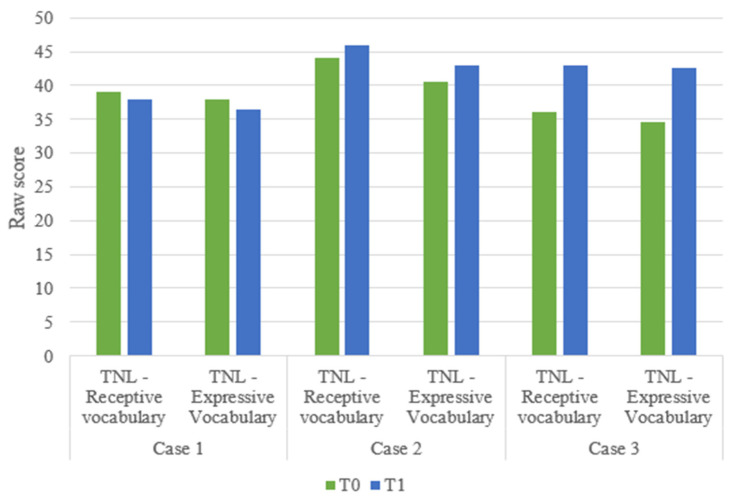
Raw scores of lexical abilities at T0 and T1.

Improvements were noted in lexical language skills, both in receptive and expressive abilities. Notably, one of the three subjects improved his lexical language skills from borderline at T0 to average at T1. One child, however, showed a slight decrease in raw scores for both receptive vocabulary (from 39 at T0 to 38 at T1) and expressive vocabulary (from 38 at T0 to 36, 5 at T1). Raw clinical assessment scores at T0 and T1 are shown in Figure 4.

**Figure 5:**
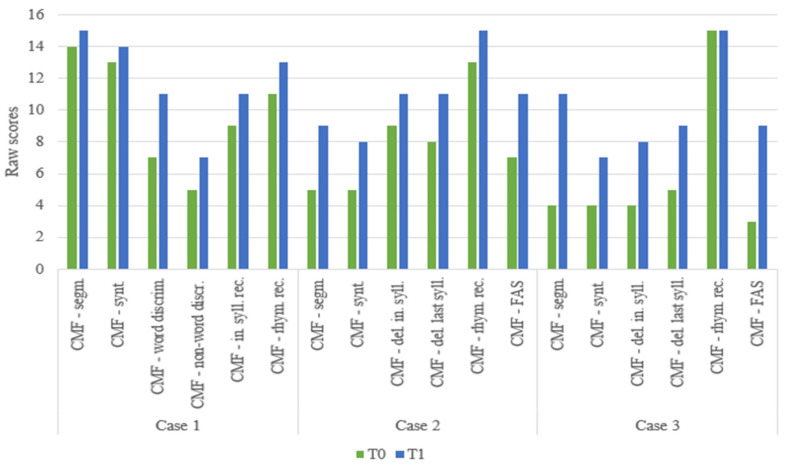
Raw scores of metaphonological abilities at T0 and T1.

Overall improvements were also observed in the metaphonological skills test scores for all three children (Figure 5).

**Figure 6:**
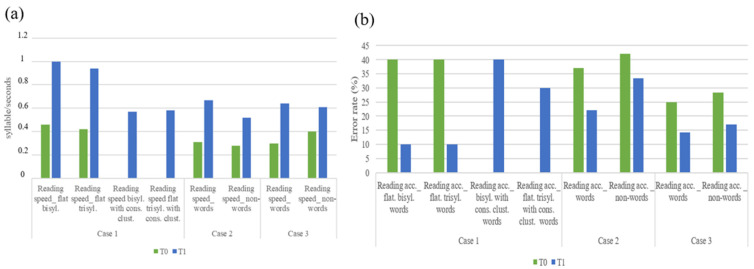
(**a**) Raw scores of reading speed at T0 and T1. (**b**) Raw scores of reading accuracy at T0 and T1.

Concerning reading abilities, all three children showed overall improvements in both speed and accuracy parameters (as shown in Figure 6). Specifically, case 1 reported an increase of 0.54 and 0.64 syll/s, respectively, in bisyllabic and trisyllabic flat words and a 75% reduction in the number of errors in both cases. Furthermore, at T1 assessment, it was possible to administer the reading tests of words with consonant clusters, which had not been feasible at T0.

Case 2 reported an increase of 0.36 and 0.24 syll/s for words and non-words, along with a 40% and 20.71% reduction in errors, respectively. Finally, case 3 showed an increase of 0.34 and 0.21 syll/s in reading speed for words and non-words, respectively, and a reduction-in-error rate of 43.20%, and 40% increase in accuracy.

**Figure 7:**
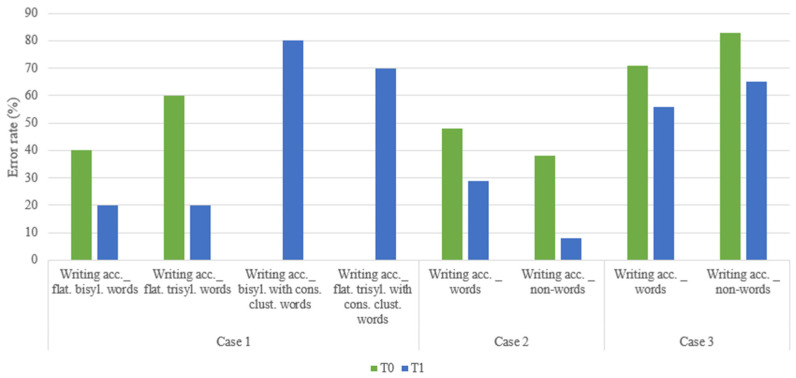
Raw scores of writing accuracy at T0 and T1.

Finally, overall improvements were also observed in writing abilities (raw scores at T0 and T1 in Figure 7).

Case 1 reported a 50% and 66.6% reduction in errors in bisyllabic and trisyllabic flat words dictation tasks. Additionally, at T1, it was possible to administer dictation tasks involving words with consonant clusters, which had not been feasible at T0.

Case 2 demonstrated a reduction-in-error percentage of 39.58% and 78% in the dictation of words and non-words, respectively, while case 3 showed a reduction of 21% and 22% in the same tasks.

## 4. Discussion

According to recent feasibility studies [41], tele-rehabilitation proved to be particularly well suited to supporting motor intervention of children with CP and their families, in order to enable continuity of care by extending rehabilitation programs into home settings, fostering family involvement with the continuous monitoring of the clinical team. However, CP often involves comorbidities, such as speech and communication disorders. This highlights the importance of exploring alternative assessment and rehabilitation methods which can address these challenges effectively.

A recent literature review [52] on the use of technology for pediatric tele-rehabilitation highlighted the lack of specific studies on their application in speech therapy for children with cerebral palsy (CP). Therefore, to the best of our knowledge, this is the first study to explore the feasibility of the virtual reality rehabilitation system (VRRS) for speech-therapy assessment in such a complex condition. Additionally, it provides preliminary data on tele-rehabilitation in a small subgroup of children with unilateral CP.

In particular, studies on VRRS application in the field of speech therapy are limited, with the initial research focusing on the adult population. Maresca et al. (2019) [28] demonstrated the effectiveness of using VRRS for language tele-rehabilitation in post-stroke adult subjects with aphasia, while Emedoli et al. (2021) [29] reported VRRS application in buccofacial apraxia treatment in an adult subject, showing overall improvements in facial movements.

Cappadona et al. (2023) [13] and Maresca et al. (2022) [27] were able to show the benefits of VRRS in pediatric speech therapy, albeit on children with dyslexia and developmental language disorders. We found that VRRS is both usable and acceptable by our stakeholders (including children with CP). We also found qualitative preliminary improvements in language and learning skills, which puts our findings in line with Cappadona et al. (2023) [13] and Maresca et al. (2022) [27].

In fact, responses to the feasibility assessment questionnaires from both clinicians and children suggest that VRRS could be a promising tool for tele-rehabilitation, demonstrating good usability and acceptability. Furthermore, qualitative analysis of the specific questionnaires’ items indicates that VRRS is an intuitive, child-friendly system well-suited for tele-rehabilitation that requires minimal technical support.

These promising results are supported by correlation analyses, which revealed no age-related limitations in the system’s application, confirming the system’s high adaptability.

However, statistically significant differences were found between the two different stakeholders both in terms of usability and acceptability, further supported by an interclass consistency analysis. In particular, children’s scores were higher than clinicians’ scores, likely due to the playful and motivating nature of the VR environment, which may be particularly attractive to younger patients, as suggested by Martins et al., 2022 [19].

The lower scores assigned by clinicians, particularly regarding acceptability, may reflect their heightened awareness of the system’s limitations. Clinicians outlined both hardware and software limitations. The most prominent hardware limitation was inconsistent touchscreen sensitivity, while software limitations included the use of unappealing images and limited customization options for certain activities (e.g., some exercises were too hard or too easy).

Therefore, as underlined by Barzacchi et al. (2024) [53], the system still requires adaptation for use in children, as confirmed by a statistically significant difference between usability and acceptability scores, highlighting the need for pediatric specific modification to better support VRRS application in clinical practice. For this reason, clinicians and technical staff must collaborate closely to ensure continuous system improvement. This involves implementing modifications to both hardware and software, informed by feasibility assessments and clinical expertise.

Investigating specifically the feasibility of using the VRRS for speech-therapy telerehabilitation in a subgroup of children with unilateral CP, overall positive results were nevertheless confirmed.

In particular, all the stakeholders had higher scores in the “system adaptability” section; responses reveal a high level of system adaptability to tele-rehabilitation, with a good success rate in treatment performance and minimal difficulties in organizing daily routines in relation to the training, confirming the potential of tele-rehabilitation to provide cost- and healthcare-time-saving for both families and the healthcare system [18].

The “Exercise Customization” section received lower scores from clinicians, according to the feasibility assessment results, which indicated the need for system adaptations, particularly in the software component. However, it is encouraging that this need originated from clinicians, who had a deeper understanding of the system’s limitations while planning the treatment, rather than from parents and children, who view tele-rehabilitation as engaging and playful.

Despite the motivating and playful environment of tele-rehabilitation, the “required effort” section received the lowest scores from both children and parents. Analysis of the responses indicates that children reported a sense of exertion after the session, while parents perceived their child as tired. Thus, although the system proves to be engaging, children perceive the activity as “exerting”. This could provide a point of reflection for treatment personalization, particularly regarding the duration of individual sessions based on the child’s characteristics, and the future integration of a tool that could assess the child’s exertion and stamina.

In addition, the adjustments proposed by clinicians in terms of exercises customization options will promote greater personalization of task complexity, potentially leading to a reduction in perceived effort.

Finally, the “appropriateness” section indicates generally positive scores; however, a qualitative analysis reveals lower ratings from clinicians, who highlight the system’s need for certain modifications, as previously noted. In contrast, higher scores were reported by parents who indicated that they could continue the tele-rehabilitation at the end of 3 months.

Considering the overall positive feasibility results of the VRRS, preliminary benefits of telerehabilitation in a subgroup of patients with unilateral CP have been explored.

All three children who attended the 3-month speech-therapy tele-rehabilitation intervention showed improvements in several areas, including lexical language skills, reading, and writing.

The most notable qualitative enhancements were observed in reading and writing skills, consistent with findings from Maresca et al. (2022) [27], which demonstrated improvements in learning abilities in children with dyslexia through VRRS rehabilitation.

In terms of reading speed, all three children demonstrated an overall increase of more than 0.3 syllables per second in word-reading tasks, indicating significant improvement beyond what usually expected from natural progression. For children with reading difficulties, the expected growth is about 0.3 syllables per second per school year for both reading text and isolated words (Tressoldi et al. (2008) [54]).

Regarding reading accuracy, participants showed a reduction in error rates ranging from 40% to 75%. This outcome approaches and, in one case, exceeds the 50% threshold identified by Tressoldi et al. (2008) [54] as the clinical criteria for determining significant improvement in a child’s reading treatment.

Reduction-in-writing error rates of 21%, 39.58%, and 50% were also observed, with two of the three children achieving results that closely approach the 50% error-reduction threshold established as the criterion for treatment effectiveness by Tressoldi et al. (2008) [54].

Despite some limitations, such as the small sample size reducing the generalizability of the findings, this study produced encouraging results about the feasibility of using VRRS to deliver speech and language activities. Future studies should involve larger cohorts to better understand the broader applicability of VRRS in speech therapy for children with CP and perform statistical analyses on the comparison of skills pre- and post-training. A larger sample could thus improve the generalizability of results, better investigating the effectiveness of tele-rehabilitation protocols in the pediatric population, for which we have laid encouraging preliminary results.

Overall, VRRS has proven to be a highly feasible and innovative tool for speech therapy in children with CP despite the complexity of this condition. Its high usability and acceptability ratings, coupled with preliminary qualitative improvements in training, support its potential integration into traditional clinical practice, despite the need of some pediatric-specific adaptations. The use of this device at home allowed for an increase in treatment frequency without additional healthcare expenses, which could explain the improvement in the evaluation metrics.

Technological systems thus represent an additional tool for clinicians, allowing the child to continue rehabilitation autonomously in offline mode, with the support of a parent and/or teacher (under clinical prescription), creating a school–home–clinician network that maintains treatment effectiveness.

Therefore, this pilot study paves the way for further research with larger sample groups which could help to evaluate the generalizability of the results and assess whether a system demonstrated to be feasible is also effective in achieving treatment outcomes.

## 5. Conclusions

This study highlights the potential advantages of the VRRS as a tele-rehabilitation tool, a user-friendly and motivating system for children that can integrate information to clinical practice and ensure continuity of care.

In particular, it is emphasized that the VRRS can be a valuable innovative system that can be used for speech-therapy rehabilitation, allowing for the enhancement of both language and learning skills in children with CP. In the case of such complex conditions as CP, the VRRS’ potential to schedule integrated interventions across multiple domains, including speech, motor, and neuropsychological functions, represents an added value in rehabilitation care.

However, the system requires some software and hardware adaptations based on suggestions provided by clinicians in order to make it more suitable for the pediatric population.

Therefore, as the application of the VRRS for pediatric speech-therapy rehabilitation is still in its early phases, further studies are necessary to advance clinical practice.

## Figures and Tables

**Table 1:** Sample characteristics.

		Subjects (*n* = 28)
Age		8.42 (12.67) years
Type of Cerebral Palsy	Spastic	25 (90%)
	Dyskinetic	3 (10%)
Sex	Female	10 (36%)
	Male	18 (64%)
Cognitive functioning	Average intellectual functioning	17 (61%)
	Intellectual disability	5 (18%)
	Borderline intellectual functioning	6 (21%)
CFCS	I	75%
	II	11%
	III	14%
GMFCS	I	18 (64%)
	II	4 (14.5%)
	III	2 (7%)
	IV	4 (14.5%)
MACS	I	9 (32%)
	II	12 (43%)
	III	7 (25%)

**Table 2:** Feasibility assessment questionnaire results.

	Clinicians (*n* = 28) Mean (SD)	Children (*n* = 22) Mean (SD)	*p*
Usability	4.29 (0.40)	4.58 (0.32)	0.031
Acceptability	3.55 (0.50)	4.42 (0.33)	0.000

**Table 3:** Feasibility-training questionnaire results.

	Clinicians Mean (SD)	Children Mean (SD)	Parents Mean (SD)
System adaptability	4.47 (0.52)	4.33 (1.07)	4.72 (0.46)
Exercises customization	3.87 (0.74)	4.22 (0.94)	4.67 (0.48)
Required effort	4.4 (0.74)	3.72 (1.36)	4.33 (0.77)
Appropriateness	4.27 (0.70)	4 (1.03)	4.33 (0.69)

## Data Availability

The datasets generated for this study are available on request to the corresponding author due to privacy reasons.

## Abbreviations

Cerebral Palsy (CP); Virtual Reality Rehabilitation System (VRRS); Specific learning disorders (SLD); Virtual Reality (VR); Manual Ability Classification System (MACS); Communication Function Classification System (CFCS); Gross motor function classification system (GMFCS); Wechsler Intelligence Scale for Children (WISC); Wechsler Preschool and Primary Scale of Intelligence (WPPSI); Technology Assessment Model (TAM); Neuropsychological Lexical Test (TNL); Assessment of Reading and Comprehension skills in Developmental Age (ALCE); Dyslexia and Developmental Dysorthography Evaluation-2 (DDE-2); Statistical Package for Social Sciences (SPSS); Intraclass correlation (ICC).

## References

[1] Bax M., Goldstein M., Rosenbaum P., Leviton A., Paneth N., Dan B., Jacobsson B., Damiano D. (2005). Proposed Definition and Classification of Cerebral Palsy, April 2005. Dev. Med. Child Neurol..

[2] Faccioli S., Sassi S., Pagliano E., Maghini C., Perazza S., Siani M.F., Sgherri G., Farella G.M., Foscan M., Viganò M. (2024). Care Pathways in Rehabilitation for Children and Adolescents with Cerebral Palsy: Distinctiveness of the Adaptation to the Italian Context. Children.

[3] Fluss J., Lidzba K. (2020). Cognitive and Academic Profiles in Children with Cerebral Palsy: A Narrative Review. Ann. Phys. Rehabil. Med..

[4] Kennes J., Rosenbaum P., Hanna S.E., Walter S., Russell D., Raina P., Bartlett D., Galuppi B. (2002). Health Status of School-aged Children with Cerebral Palsy: Information from a Population-based Sample. Dev. Med. Child Neuro.

[5] Mei C., Reilly S., Reddihough D., Mensah F., Pennington L., Morgan A. (2016). Language Outcomes of Children with Cerebral Palsy Aged 5 Years and 6 Years: A Population-based Study. Dev. Med. Child Neuro.

[6] Fiori S., Ragoni C., Podda I., Chilosi A., Amador C., Cipriani P., Guzzetta A., Sgandurra G. (2022). PROMPT to Improve Speech Motor Abilities in Children with Cerebral Palsy: A Wait-List Control Group Trial Protocol. BMC Neurol..

[7] Jenks K.M., Van Lieshout E.C.D.M., De Moor J.M.H. (2012). Cognitive Correlates of Mathematical Achievement in Children with Cerebral Palsy and Typically Developing Children. Br. J. Educ. Psychol..

[8] Colver A., Rapp M., Eisemann N., Ehlinger V., Thyen U., Dickinson H.O., Parkes J., Parkinson K., Nystrand M., Fauconnier J. (2015). Self-Reported Quality of Life of Adolescents with Cerebral Palsy: A Cross-Sectional and Longitudinal Analysis. Lancet.

[9] Wotherspoon J., Whittingham K., Sheffield J., Boyd R.N. (2023). Cognition and Learning Difficulties in a Representative Sample of School-Aged Children with Cerebral Palsy. Res. Dev. Disabil..

[10] Pennington L., Goldbart J., Marshall J. (2004). Speech and Language Therapy to Improve the Communication Skills of Children with Cerebral Palsy. Cochrane Database Syst. Rev..

[11] Pennington L., Cunningham S., Hiu S., Khattab G., Ryan V. (2023). The Impact of the Speech Systems Approach on Intelligibility for Children with Cerebral Palsy: A Secondary Analysis. Effic. Mech. Eval..

[12] Lee S.A.S., Hall B., Sancibrian S. (2017). Feasibility of a Supplemental Phonological Awareness Intervention via Telepractice for Children with Hearing Loss: A Preliminary Study. Int. J. Telerehabilitation.

[13] Cappadona I., Ielo A., La Fauci M., Tresoldi M., Settimo C., De Cola M.C., Muratore R., De Domenico C., Di Cara M., Corallo F. (2023). Feasibility and Effectiveness of Speech Intervention Implemented with a Virtual Reality System in Children with Developmental Language Disorders: A Pilot Randomized Control Trial. Children.

[14] Saine N.L., Lerkkanen M.-K., Ahonen T., Tolvanen A., Lyytinen H. (2011). Computer-Assisted Remedial Reading Intervention for School Beginners at Risk for Reading Disability: Computer-Assisted Reading Intervention. Child Dev..

[15] Sarti D., De Salvatore M., Gazzola S., Pantaleoni C., Granocchio E. (2020). So Far so Close: An Insight into Smart Working and Telehealth Reorganization of a Language and Learning Disorders Service in Milan during COVID-19 Pandemic. Neurol. Sci..

[16] Pecini C., Spoglianti S., Bonetti S., Di Lieto M.C., Guaran F., Martinelli A., Gasperini F., Cristofani P., Casalini C., Mazzotti S. (2019). Training RAN or Reading? A Telerehabilitation Study on Developmental Dyslexia. Dyslexia.

[17] Keck C.S., Doarn C.R. (2014). Telehealth Technology Applications in Speech-Language Pathology. Telemed. e-Health.

[18] Cantagallo A. (2014). Teleriabilitazione e Ausili: La Tecnologia in Aiuto Alla Persona Con Disturbi Neuropsicologici.

[19] Martins S., Cavaco S., Otero P., Scott P., Martin S.Z., Huesing E. (2022). Customizable Serious Speech Therapy Games with Dynamic Difficulty Adjustment for Children with Sigmatism. Studies in Health Technology and Informatics.

[20] Dostie R., Gaboury I., Cinar E., Camden C. (2022). Acceptability of Pediatric Telerehabilitation Interventions Provided by Physical Therapists and Occupational Therapists—A Scoping Review. Phys. Occup. Ther. Pediatr..

[21] Kizony R., Katz N., (Tamar) Weiss P.L. (2003). Adapting an Immersive Virtual Reality System for Rehabilitation. J. Visual. Comput. Animat..

[22] Macchitella L., Amendola S., Barraco G., Scoditti S., Gallo I., Oliva M.C., Trabacca A. (2023). A Narrative Review of the Use of a Cutting-Edge Virtual Reality Rehabilitation Technology in Neurological and Neuropsychological Rehabilitation. NeuroRehabilitation.

[23] Gamito P., Oliveira J., Coelho C., Morais D., Lopes P., Pacheco J., Brito R., Soares F., Santos N., Barata A.F. (2017). Cognitive Training on Stroke Patients via Virtual Reality-Based Serious Games. Disabil. Rehabil..

[24] You S.H., Jang S.H., Kim Y.-H., Hallett M., Ahn S.H., Kwon Y.-H., Kim J.H., Lee M.Y. (2005). Virtual Reality–Induced Cortical Reorganization and Associated Locomotor Recovery in Chronic Stroke: An Experimenter-Blind Randomized Study. Stroke.

[25] Kim O., Pang Y., Kim J.-H. (2019). The Effectiveness of Virtual Reality for People with Mild Cognitive Impairment or Dementia: A Meta-Analysis. BMC Psychiatry.

[26] Zhao J.-Q., Zhang X.-X., Wang C.-H., Yang J. (2021). Effect of Cognitive Training Based on Virtual Reality on the Children with Autism Spectrum Disorder. Curr. Res. Behav. Sci..

[27] Maresca G., Leonardi S., De Cola M.C., Giliberto S., Di Cara M., Corallo F., Quartarone A., Pidalà A. (2022). Use of Virtual Reality in Children with Dyslexia. Children.

[28] Maresca G., Maggio M.G., Latella D., Cannavò A., De Cola M.C., Portaro S., Stagnitti M.C., Silvestri G., Torrisi M., Bramanti A. (2019). Toward Improving Poststroke Aphasia: A Pilot Study on the Growing Use of Telerehabilitation for the Continuity of Care. J. Stroke Cerebrovasc. Dis..

[29] Emedoli D., Arosio M., Tettamanti A., Iannaccone S. (2021). Virtual Reality Augmented Feedback Rehabilitation Associated to Action Observation Therapy in Buccofacial Apraxia: Case Report. Clin. Med. Insights Case Rep..

[30] Eliasson A.-C., Krumlinde-Sundholm L., Rösblad B., Beckung E., Arner M., Öhrvall A.-M., Rosenbaum P. (2006). The Manual Ability Classification System (MACS) for Children with Cerebral Palsy: Scale Development and Evidence of Validity and Reliability. Dev. Med. Child Neurol..

[31] Hidecker M.J.C., Paneth N., Rosenbaum P.L., Kent R.D., Lillie J., Eulenberg J.B., Chester Jr K., Johnson B., Michalsen L., Evatt M. (2011). Developing and Validating the Communication Function Classification System for Individuals with Cerebral Palsy: Developing a Communication Classification System. Dev. Med. Child Neurol..

[32] Palisano R.J., Rosenbaum P., Bartlett D., Livingston M.H. (2008). Content Validity of the Expanded and Revised Gross Motor Function Classification System. Dev. Med. Child Neuro.

[33] Dade P., Goldstein S., Naglieri J.A. (2011). Encyclopedia of Child Behavior and Development.

[34] Caplan B., DeLuca J., Kreutzer J.S., Caplan B., DeLuca J. (2011). Encyclopedia of Clinical Neuropsychology.

[35] Roid G.H., Cornoldi C. (2022). Leiter-3 Leiter International Performance Scale [Valigia].

[36] Piron L., Turolla A., Agostini M., Zucconi C., Tonin P., Piccione F., Dam M. (2009). Assessment and Treatment of the Upper Limb by Means of Virtual Reality in Post-Stroke Patients. Stud. Health Technol. Inform..

[37] Agostini M., Moja L., Banzi R., Pistotti V., Tonin P., Venneri A., Turolla A. (2015). Telerehabilitation and Recovery of Motor Function: A Systematic Review and Meta-Analysis. J. Telemed. Telecare.

[38] Olivieri I., Chiappedi M., Meriggi P., Mazzola M., Grandi A., Angelini L. (2013). Rehabilitation of Children with Hemiparesis: A Pilot Study on the Use of Virtual Reality. BioMed Res. Int..

[39] Beani E., Filogna S., Martini G., Barzacchi V., Ferrari A., Guidi E., Menici V., Cioni G., Sgandurra G. (2022). Application of Virtual Reality Rehabilitation System for the Assessment of Postural Control While Standing in Typical Children and Peers with Neurodevelopmental Disorders. Gait Posture.

[40] Martini G., Beani E., Filogna S., Menici V., Cioni G., Battini R., Sgandurra G. (2022). New Technological Approach for the Evaluation of Postural Control Abilities in Children with Developmental Coordination Disorder. Children.

[41] Menici V., Barzacchi V., Filogna S., Beani E., Tinelli F., Cioni G., Sgandurra G. (2021). Tele-Rehabilitation for Postural Control by Means of Virtual Reality Rehabilitation System in an Adolescent With Motor Disorder: A Case Study. Front. Psychol..

[42] Sgherri G., Avola M., Beani E., Chisari C., Cioni G., Sgandurra G. (2019). Methods to Assess Usability and Acceptability of Technologies for Home-Based Rehabilitation a Systematic Review. Int. J. Emerg. Technol..

[43] Wixon D., Wilson C. (1997). The Usability Engineering Framework for Product Design and Evaluation. Handbook of Human-Computer Interaction.

[44] Jokela T., Iivari N., Matero J., Karukka M. The Standard of User-Centered Design and the Standard Definition of Usability: Analyzing ISO 13407 against ISO 9241-11. Proceedings of the Latin American Conference on Human-Computer Interaction—CLIHC ’03.

[45] Abran A., Khelifi A., Suryn W., Seffah A. (2003). Usability Meanings and Interpretations in ISO Standards. Softw. Qual. J..

[46] Davis F.D. (1989). Perceived Usefulness, Perceived Ease of Use, and User Acceptance of Information Technology. MIS Q..

[47] Cossu G. (2013). TNL—Test Neuropsicologico Lessicale Per l’età Evolutiva.

[48] Marotta L., Ronchetti C., Trasciani M., Vicari S. (2008). CMF: Valutazione Delle Competenze Metafonologiche.

[49] Bonifacci P., Tobia V., Lami L., Snowling M.J. (2014). ALCE. Assessment Di Lettura e Comprensione in Età Evolutiva Assessment of Reading and Comprehension in Developmental Age.

[50] Martini A. (2004). Le Difficoltà di Apprendimento Della Lingua Scritta: Criteri di Diagnosi e Indirizzi di Trattamento.

[51] Sartori G., Job R., Tressoldi P.E. (2013). DDE-2: Batteria per La Valutazione Della Dislessia e Della Disortografia Evolutiva-2: Manuale.

[52] Del Lucchese B., Parravicini S., Filogna S., Mangani G., Beani E., Di Lieto M.C., Bardoni A., Bertamino M., Papini M., Tacchino C. (2024). The Wide World of Technological Telerehabilitation for Pediatric Neurologic and Neurodevelopmental Disorders—A Systematic Review. Front. Public Health.

[53] Barzacchi V., Mangani G., Del Lucchese B., Menici V., Bombonato C., Beani E., Biagioni E., Palla I., Posteraro F., Trieste L. (2024). TABLET TOSCANA to Develop Innovative Organizational Models for Tele-Rehabilitation in Subjects with Congenital and Acquired Developmental Disabilities: A Wait-List Control Group Trial Protocol. J. Clin. Med..

[54] Tressoldi P.E., Vio C. (2008). Significatività clinica negli studi di efficacia dei trattamenti per i disturbi dell’apprendimento: Una proposta. Psicol. Clin. Svilupp..

